# Radiation transmission, leakage and beam penumbra measurements of a micro‐multileaf collimator using GafChromic EBT film

**DOI:** 10.1120/jacmp.v9i3.2802

**Published:** 2008-06-23

**Authors:** Olivia Amanda García‐Garduño, Miguel Ángel Celis, José Manuel Lárraga‐Gutiérrez, Sergio Moreno‐Jiménez, Arnulfo Martínez‐Dávalos, Mercedes Rodríguez‐Villafuerte

**Affiliations:** ^1^ Laboratorio de Física Médica y Unidad de Radioneurocirugía Instituto Nacional de Neurología y Neurocirugía México D. F. México; ^2^ Instituto de Física Universidad Nacional Autónoma de México México D. F. México

**Keywords:** mMLC, MLC transmission, MLC penumbra, GafChromic, dosimetry, IMRT

## Abstract

Micro‐multileaf collimator systems coupled to linear accelerators for radioneurosurgery treatments require a rigorous dosimetric characterization in order to be used in 3D conformal and intensity modulated stereotactic radiosurgery and radiotherapy applications. This characterization involves high precision measurements of leaf transmission, leakage and beam penumbra through the collimation system and requires the use of detectors with high spatial resolution, high sensitivity and practically no energy dependence. In this work the use of GafChromic EBT radiochromic film to measure the basic dosimetric properties of the m3‐mMLC (BrainLAB, Germany) micro‐multileaf collimator system integrated to a 6 MV linear accelerator, is reported. Results show that average values of transmission and leakage radiation are 0.93±0.05% and 1.08±0.08%, respectively. The 80–20% beam penumbra were found to be 2.26±0.11 mm along the leaf side (perpendicular to leaf motion) and 2.31±0.11 mm along the leaf end (parallel to leaf motion) using square field sizes ranging from 9.1 to 1.8 cm. These measurements are in agreement with values reported in the literature for the same type of mMLC using different radiation detectors.

PACS number: 87.56.N‐

## I. INTRODUCTION

Multileaf collimators (MLCs) are beam modifiers coupled to medical linear accelerators to shape the photon beam in order to match the geometry of the lesion to be irradiated. The general structure of these devices consists of a set of leaves generally made of a tungsten alloy. The position of the leaves is individually controlled by a computer, allowing their movement along a direction perpendicular to the beam axis. A variation of this device is the so‐called micro‐MLC (mMLC). The main difference between standard MLCs and mMLCs is the leaf width. Typically, a standard MLC has leaf widths ranging from 0.5 to 1.0 cm, while an mMLC has leaf widths ranging from 0.1 to 0.4 cm. Current mMLCs are designed for the treatment of lesions smaller than 8.0 cm.

The use of several detectors, such as small ionization chambers and radiographic films (X‐OMAT V2 and EDR2) for the characterization of basic dosimetric properties of mMLC systems, such as leaf transmission, leakage and beam penumbra measurements, has been reported extensively in the literature.^1^, ^6^ Small ionization chambers provide high precision measurements for field sizes larger than their effective active area. However, due to their finite size, their response might present partial volume effects for small beam measurements.^7^, ^8^ On the other hand, although radiographic films have high spatial resolution, they present high energy dependence, producing an over‐response to low energy scattered photons, which results in an overestimation of beam penumbra.^(7)^

The use of radiochromic films in radiotherapy, such as MD‐55–2, has been reported previously,^(9)^ however, this film has low sensitivity and its use is difficult for dose measurements below 3.0 Gy.^(10)^ The introduction of GafChromic EBT film (International Specialty Products, New Jersey, USA) during the last years has overcome all of these problems. They are particularly attractive for dosimetric applications because their chemical composition is nearly tissue equivalent producing limited energy dependence when compared to radiographic films.^(11)^ Additionally, their high spatial resolution and dynamic range (1–800 cGy) allows a high level of accuracy for dose assessment in penumbral regions. In this work we report experimental measurements of radiation transmission, leakage and 80–20% beam penumbra of the m3‐mMLC (BrainLAB, Germany) micro‐multileaf collimator system coupled to a 6 MV linear accelerator using GafChromic EBT radiochromic film, and compare our results with those reported previously using other type of detectors.

## II. MATERIALS AND METHODS

### A. GafChromic EBT films

Radiochromic phenomena involve the direct coloration of a material by the absorption of radiation without the use of external chemical, optical or thermal agents. Radiochromic films are nearly transparent before irradiation and consist of a micro‐crystalline active thin layer based on polydiacetylene, coated on a flexible polyester film base. After irradiation, their color changes to blue due to polymerization effects.^(10)^

The internal structure of GafChromic EBT consists of two active layers of 17 μm thickness separated by a 6 μm layer coated on a 97 μm polyester base on each side. This design decreases the effects of environmental and ultraviolet light.^(7)^ The atomic composition of the film material is C (42.3%), H (39.7%), O (16.2%), N (1.1%), Li (0.3%) and Cl (0.3%) with an effective atomic number of 6.98. Its sensitivity is 10 times larger than its predecessors, such as GafChromic MD‐55–2 and HS. The useful dose range is from 1 to 800 cGy, and presents two absorption peaks at 636 and 585 nm,^(12)^ its response is energy independent,^(13)^ and according to the manufacturer specifications, it has a real time response.

In this work, all the measurements were performed using GafChromic EBT film with serial number 36124–003I. The films were carefully handled and analyzed following the recommendations of the TG55 report^(10)^ and those proposed by Butson and co‐workers.^(14)^ The films were placed in light‐protecting envelopes, and were only removed from them during irradiation and readout, to reduce the effects of ambient light. Moreover, at all times the ambient lights were dimmed in order to avoid stray light effects. The un‐irradiated films were scanned 24 h previous to irradiation and the readout was performed 48 h after irradiation, taking care of maintaining the same orientation and placement on the scanner glass tray. During the storage, irradiation, and readout the films were kept at a temperature of 21 °C and 50% humidity controlled by the cooling system of our laboratory.

### B. Data analysis and processing

Readout of the EBT films was performed with a commercial flatbed scanner ScanMaker 9600XL (Microtek, USA) working in transmission mode. This scanner has a maximal optical spatial resolution of 1200×600 dpi and 36‐bit color depth (12‐bits per channel: red, green and blue).

Films were scanned using the commercial software FileScan v3.09 (Microtek, USA) with 36‐bits color depth and 100 dpi, with all post‐processing and color management options turned off. The images were stored in tagged image file format (TIFF) and analyzed using ImageJ.^(15)^ Only the red component of the film response was used for the analysis, due to its higher sensitivity as compared with the green and blue components.^(16)^

In order to correct for non‐uniformities caused by the light scattering of the scanner lamp^(17)^ and intrinsic heterogeneities on the film structure, an image background correction was applied following a procedure suggested by the film manufacturer/^18^) The films were digitized twice, before and after irradiation. The non‐irradiated film image $\left(\varphi_{o}\right)$ was normalized with respect to its mean background value calculated over the entire film surface. The irradiated image $\left(\varphi_{i}\right)$ was then corrected for non‐uniformities using:

(1) $$\varphi_{c}=\frac{\varphi_{i}}{{\hat{\varphi }}_{o}}$$

where ${\hat{\varphi }}_{o}$ and $\varphi_{c}$ correspond to the normalized and the corrected images, respectively. In order to warrant that the pre‐ and post‐ irradiation images were spatially registered, each film was marked with a fiducial and placed in the same position and orientation in the scanner glass tray. Film response was measured using the following expression^(16)^:

(2) $$R=\log\left(\frac{\varphi_{o}}{\varphi_{c}}\right)$$

### C. Novalis m3‐mMLC collimation system

**Figure 1:**
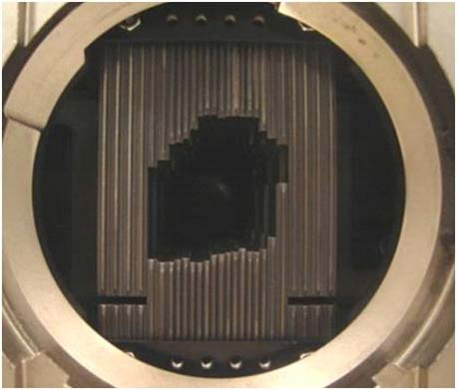
Photograph of the m3‐MLC Novalis.

Measurements were performed using a dedicated Novalis linear accelerator (BrainLAB, Gmbh, Germany) based on a Varian 600N (Varian Associates, USA) working on a 6 MV photon beam mode. An m3 model micro‐MLC system (BrainLAB, Gmbh, Germany and Varian Associates, USA) is integrated to the linac (see Fig. 1). The m3‐mMLC has 26 pairs of tungsten alloy (95% W, 3.4% Ni and 1.6% Fe) leaves of several widths, as shown in Table 1.

**Table 1:** Design characteristics of the m3‐MLC according to the manufacturer specifications.

Number of leaves	26 pairs
Leaf width (at isocenter)	14×3.0 mm
	6×4.5 mm
	6×5.5 mm
Maximum field	10.2 cm
Maximum useful field	$9.8\times 9.8\;{\text{cm}}^{2}$
Maximum leaf over‐travel	10.0 cm
Maximum leaf velocity	1.5 cm/s
Weight	30 kg

### D. Film calibration

Film calibration was performed by placing the film on a polymethy 1 methacrylate (PMMA, 1.15 g/cm^3^ density) phantom consisting of 6 slabs with total dimensions of $20\times 20\times 30\;{\text{cm}}^{3}$. The film was placed at 2.22 cm depth (equivalent to 2.5 cm in water) and irradiated using the SSD technique (SSD=100 cm). The film was irradiated with a field size of $3\times 3\;{\text{cm}}^{2}$ covering a dose range between 1 to 450 cGy. Absolute doses were measured following the IAEA TRS 277 formalism^(19)^ using a 0.125 cm^3^ PTW‐31010 semiflex ionization chamber (PTW‐Freiburg, Germany) placed at the same depth as the film within a special slab drilled to fit the chamber dimensions.

### E. Film irradiation

Radiation transmission, leakage and 80–20% beam penumbra were measured by placing the film perpendicular to the beam central axis at a depth of 1.3 cm $\left(d_{\max}\right)$ in the PMMA phantom (1.5 cm water equivalent) using the SAD (98.7 cm SSD) technique.

Five measurements of the 80–20% beam penumbra were performed using 250 monitor units (MU) and 5 different field sizes: 9.1×9.1, 8.0×8.0, 6.0×6.0, 4.2×4.2 and $1.8\times 1.8\;{\text{cm}}^{2}$. For each field size, an average 80–20% beam penumbra was obtained using 10 horizontal profiles at different positions in the irradiated film.

Transmission and leakage radiation measurements were carried out placing the film under the same irradiation conditions as for the beam penumbra. Two irradiations were performed: one with 200 MU using an open field of $9.8\times 9.8\;{\text{cm}}^{2}$ defined by the mMLC, and another with 3000 MU and the mMLC closed, completely blocking the photon beam. The MU used for both irradiations were selected such that the film response was within the dose interval used in the calibration curve. The measured doses in the irradiated film when using a blocked field were normalized by a factor of 15 (3000/200) in order to compensate for the differences in photon flux at the mMLC entrance. The transmitted radiation through the mMLC was estimated as a percentage according to:

(3) $$T(\%)=\frac{D_{blocked}}{D_{open}}\times 100$$

where $D_{\text{blocked}}$ and $D_{\text{open}}$ represent horizontal dose profiles across the blocked and open fields, respectively.

## III. RESULTS

**Figure 2:**
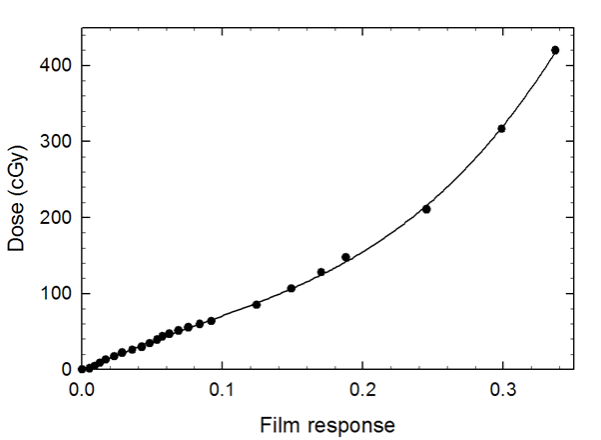
GafChromic EBT calibration curve in the dose range from 1 to 450 cGy. The solid curve shows the cubic fit.

Fig. 2 shows the calibration curve of the EBT film in the 0–450 cGy dose interval. A cubic fit over the whole dose interval produced the following expression:

(4) $$D(R)=-2.72+922.58R-3058.82R^{2}+11944.54R^{3}$$

where *D* is the dose in cGy and *R* is the film response according to equation 2. All dose measurements reported from here onwards were obtained from the digitized response measurements using equation 4.

**Figure 3:**
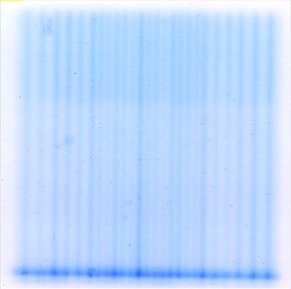
Image of the GafChromic EBT film used for transmission and leakage radiation measurements (blocked field).

**Figure 4:**
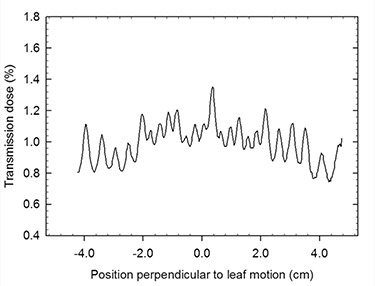
Transmission dose profile obtained from and Equation 4. The valleys correspond to photon transmission through the leaves and the peaks to leakage radiation.

Fig. 3 shows the digitized EBT film obtained with the mMLC closed when using 3000 MU during transmission and leakage radiation measurements. A non uniform strip pattern can be observed, corresponding to radiation transmission (low intensity) and leakage (high intensity). A horizontal dose profile extracted from this image is shown in Fig. 4. The peaks and valleys in this profile correspond to radiation leakage and transmission through the leaves, respectively. The overall doses are larger in the centre of the profile due to differences in the leaf widths (the outer leaves are wider than the central ones). Average values of 0.93±0.05% for transmission and 1.08±0.08% for radiation leakage were obtained. Table 2 shows the average values of these quantities for different field regions.

**Table 2:** Average values of transmission and leakage radiation for the central and peripheral mMLC regions.

*Region*	*Transmission (%)*	*Leakage (%)*
Central	1.01±0.04	1.13±0.08
Periphery	0.85±0.07	1.04±0.07

**Figure 5:**
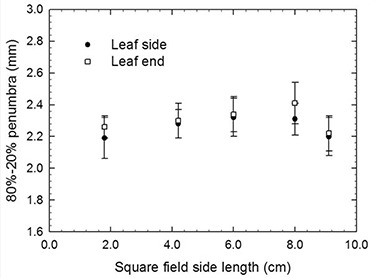
m3‐mMLC 80–20% beam penumbra for square fields.

The 80–20% beam penumbra as a function of field size is shown in Table 3 and plotted in Fig. 5. The 80–20% beam penumbrae averaged over all field sizes were 2.26±0.11 mm along the leaf side (perpendicular to leaf motion) and 2.31±0.11 mm along the leaf end (parallel to leaf motion).

**Table 3:** m3‐mMLC 80–20% beam penumbra as a function of field size measured perpendicular (leaf side) and parallel (leaf end) to leaf motion.

	*Penumbra 80–20% (mm)*	
*Field size (cm* ^2^)	*Leaf side*	*Leaf end*
9.1×9.1	2.20±0.12	2.22±0.11
8.0×8.0	2.31±0.10	2.41±0.13
6.0×6.0	2.32±0.12	2.34±0.11
4.2×4.2	2.28±0.09	2.30±0.11
1.8×1.8	2.19±0.13	2.26±0.07
Average	2.26±0.11	2.31±0.11

## IV. DISCUSSION

By following the protocol for the handling and analysis of the EBT films as described in section 2, the overall one‐sigma dose measurement uncertainty for a uniform field amounts to 4% or less for doses above 40 cGy. The reproducibility of the measurements reported in this work has been evaluated over a one year period and found to be within 4%. It is known that these values are specific for the type of film, digitizer and dose range used.^17^, ^20^ In our case a document scanner was used, and a similar system used with EBT film reported overall dose uncertainties below 6% at approximately 1 Gy for a similar (single set/single scan) analysis procedure.^(20)^

Table 4 compares the transmission and leakage radiation measurements for the m3‐mMLC obtained in this work with those reported in the literature for the same type of mMLC when using different detectors. As a reference, a couple of values for the same nominal energy and different collimator manufacturer are included in the table. It can be observed that our values are consistently lower than all the previously reported measurements using the m3‐mMLC.

**Table 4:** mMLC transmission and leakage radiation values reported in the literature. A 6 MV photon beam was used in all cases.

*Collimator*	*Transmission (%)*	*Leakage (%)*	*Detector*
m3‐MLC (This work)	0.93±0.05	1.18±0.11	GafChromic EBT
m3‐MLC (Belec 2005)	1.3	2.4	Kodak X‐OMAT V2
m3‐MLC (Aaronson 2002)	1.3	2.1	Kodak EDR2
m3‐MLC (Agazaryan 2000)	1.53	2.10	Kodak X‐OMAT V2
m3‐MLC (Cosgrove 1999)	1.9±0.1	2.8±0.15	Kodak X‐OMAT V2
Radionics mMLC (Wang 2006)	0.85	1.4	Ionization chamber
Radionics mMLC (Mardirossian 2003)	0.9±0.06	1.3±0.10	Kodak X‐OMAT V2

It is important to note that the values vary according to the laboratory and the type of detector used. However, a trend of improving performance with time can be observed which might be due to corresponding improvements in the manufacturing of the mMLC leaves, and the use of better techniques and detectors to measure these properties.

Measurements of the 80–20% beam penumbra along the leaf side for the five field sizes presented minimum and maximum values of 2.19 and 2.32 mm for the 1.8×1.8 and $6.0\times 6.0\;{\text{cm}}^{2}$ fields, respectively. Along the leaf end these values were 2.22 and 2.41 mm for the 9.1×9.1 and $8.0\times 8.0\;{\text{cm}}^{2}$ fields, respectively. These values are slightly smaller than those reported by Cosgrove et al.^(1)^ (2.45 mm perpendicular to leaf motion and 2.60 mm parallel with leaf motion) for square fields when using radiographic film X‐OMAT V2. This might be attributed to the energy dependence of the radiographic film which produces broadening of penumbra measurements.^(7)^

## V. CONCLUSIONS

In this work the use of GafChromic EBT radiochromic film to measure transmission, leakage and beam penumbra of the BrainLAB m3‐mMLC micro‐multileaf collimator is reported. Results show that average values of transmission and leakage radiation are 0.93±0.07% and 1.08±0.08%, respectively. The 80–20% beam penumbra were found to be 2.26±0.11 mm along the leaf side and 2.31±0.11 mm along the leaf end using square field sizes ranging from 9.1 to 1.8 cm. These measurements are in agreement with those reported in the literature using different radiation detectors.

The results on this work show that some of the basic dosimetric properties of the m3‐mMLC for stereotactic radiosurgery and radiotherapy applications can be measured with high precision using GafChromic EBT radiochromic film. These films have several advantages when compared to other detectors, namely, they have high spatial resolution and sensitivity, their response is independent of the photon energy, and their readout can be performed with relatively cheap equipment. However, it is important to use a very strict protocol for the handling and readout of the films if reproducible and high precision results are to be obtained.

## ACKNOWLEDGMENTS

This work was partially supported by Conacyt U46761‐F.
